# Supplementary material for: Fabrication of CuO-NP-Doped PVDF Composites Based Electrospun Triboelectric Nanogenerators for Wearable and Biomedical Applications
Source: Polymers (Basel). 2023 May 25;15(11):2442. doi: 10.3390/polym15112442 (PMC10255730; doi:10.3390/polym15112442)
Supplement: Supplementary file 1 [file polymers-15-02442-s001.zip › polymers-2283355-supplementary.pdf]

Supplementary Materials: Fabrication of CuO-NP-Doped PVDF Composites Based Electrospun Triboelectric Nanogenerators for Wearable and Biomedical Applications

Bindhu Amrutha [1], Gajula Prasad [2], Ponnan Sathiyanathan [3,†], Mohammad Shamim Reza [3], Hongdoo Kim [3], Madhvesh Pathak [1,*] and Arun Anand Prabu [1,*]

1 Department of Chemistry, School of Advanced Sciences, Vellore Institute of Technology, Vellore 632014, India
2 School of Energy, Materials and Chemical Engineering, Korea University of Technology and Education, 1600, Cheonan-si 31253, Republic of Korea
3 Department of Advanced Materials Engineering for Information & Electronics, College of Engineering, Kyung Hee University, Yongin-si 17104, Gyeonggi-do, Republic of Korea
* Correspondence: madhveshpathak@vit.ac.in (M.P.); anandprabu@vit.ac.in (A.A.P.)
† Present address: Department of Mechanical Engineering, Korea Advanced Institute of Science and Technology (KAIST), Daejeon 34141, Republic of Korea.

Supplementary Material

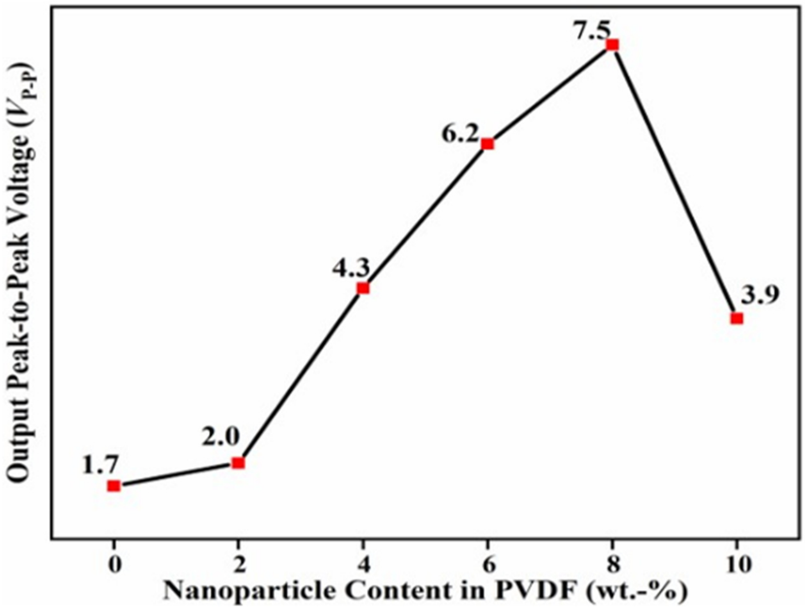

Figure S1. Quantitative graph of peak-to-peak voltage of TENGs with various wt.-% of CuO NPs

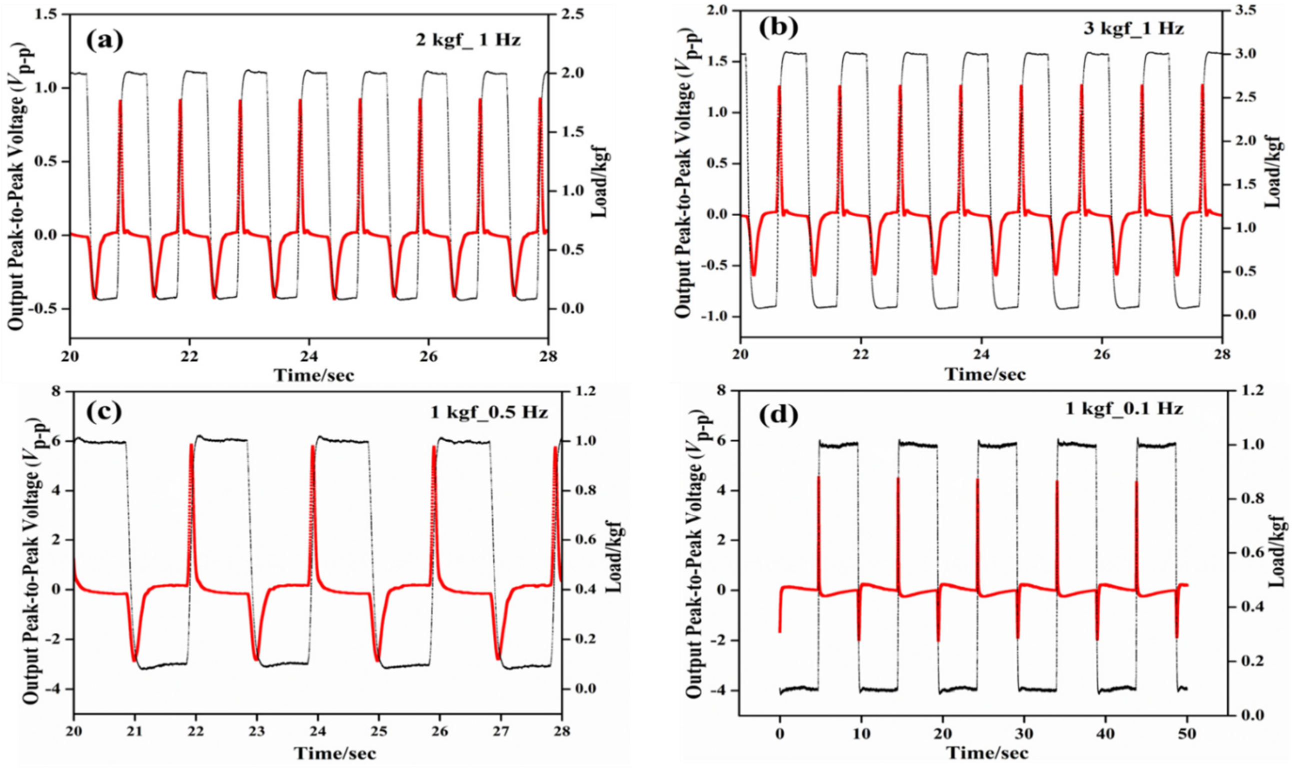

Figure S2. Output voltage of PC-8/PU TENG measured under (a) 2 kgf and 1 Hz; (b) 3 kgf and 1 Hz; (c) 1 kgf and 0.5 Hz and (d) 1 kgf and 0.1 Hz.
